# A Plea for the "Practical" Man

**Published:** 1892-10-08

**Authors:** 


### The Hospital

An Institutional Journal of

Science, Medicine, Nursing, and Philanthropy.

*For Hospitals, Asylums, Infirmaries, Dispensaries, Medical Practitioners, Students, Nurses, and the Charitable Public.*

Vol. XIII.—No. 315.] [Oct. 8, 1892.

### A Plea for the "Practical" Man.

Dr. R. L. Bowles, at the opening of the St. George's Hospital session on Saturday, cast another stone at the "so called practical man." There have been so many stones thrown at that unfortunate person by physicians and surgeons who themselves, it must be assumed, prefer to be, and to be called, "unpractical," that he must by this time be buried beneath a whole cairn of them. Happily, he still survives.

Everybody has heard of the "Avocatus diaboli," and the poet Burns went so far as to express the hope that even so hardened an offender as Diabolus himself might some time or other "Tak a thocht and mend." We only venture into this non-medical region for a single moment for the purpose of pointing out that the "practical" doctor can by no means be in such an abandoned condition as the father of lies; and that therefore it is permissible to enter at least a protest, and perhaps even a plea, on his behalf. Since we are dealing with scientific men, it will be felt to be quite proper that we should ask those who object to the "practical" man to consider the subject scientifically. And, first, What is it that Dr. Bowles and others like-minded with himself exactly mean by the "so-called practical man," of whom they speak so derisively? It is to be presumed that they mean the ordinary family practitioner, whose work is so arduous that he has not time and strength to keep himself so fully abreast as he could wish with the latest developments in scientific medicine.

Let it be admitted that there are practitioners who, with the best intentions in the world, find themselves unable to quite keep abreast of all the latest theories in pathology and the most recent methods in the medical art. Do such men deserve in any sense to be held up to ridicule? and, above all, to the ridicule of medical students, many of whom are merely first year's men? So far from this being the case, we claim for the hard-worked family practitioner that it is his greatest honour that he is "practical." Nay, more; we affirm that in being practical he is carrying out in his measure the methods of the scientific laboratory, of the hospital clinique, and, in short, of the most advanced science of the times. What is the one distinguishing mark and feature of modern, as compared with mediæval medical science? It is the fact of experimentation, of stern "practicality." The laboratory is the place of experiment: the place where old theories are corrected by experiment, and new theories are based upon experiment: the place where every theory stands or falls by its proved "unpracticality" or "practicality." What, too, is the one prominent note of the hospital clinique? Here, again, it is experiment, and evermore experiment: Experiment upon human creatures succeeding the "animal" experiments of the laboratory. What are the vivisectors constantly telling an over-suspicious world? They are iterating and reiterating that modern medicine is based and built, brick by brick, and roofed-in, and crowned with its topstone, by experiment and experiment alone; by, in fact, the most entirely practical of all methods which the human mind has ever devised.

But, all these facts notwithstanding, it is the lights of the laboratory and the clerks of the clinique who turn upon the sternly toiling practitioner and abuse him because he is "practical." The case is positively exasperating in its absurdity. If the experimenters of the laboratory and the clinique had charged the average practitioner with being "unpractical," and had, therefore, held him up to ridicule, we could have understood it; and a very serious charge it would have been. But to make it a charge of heinous unworthiness against a man that he is "practical," is like charging a newly-washed sheep with filthiness because its fleece is white, or a Cæsar's wife with infidelity because she is above suspicion.

Let us consider what part it is that the average practitioner actually does play in the onward march and progress of medical science. Is he a mere drudge and slave in the great army? Too often, it is to be feared, he is both a drudge and a slave; but that is not the ordinary function of the "so-called practical man." His function is like that of the general reading public in questions of literature. The author writes his book, and the critic says his say upon it. But who is it that determines its real fate and final place in literature? It is the average reading public, the mere "practical" men! It is exactly the same with medicine and its progress. The man in the laboratory makes his discovery; his fellow-scientist in the hospital clinique proceeds with his theory and hispractice—his practice, *mirabile dictu!*—and he tries his theory upon patient after patient by the experimental methods of the practical man. But when the laboratory scientist and the hospital clinician have done and completed their work, what is the next step in the scientific process? They publish in the medical journals; they make their appeal to the mere general practitioner, to the despised practical man; and, can it be really believed! it is this very man who ultimately settles the fate of every new method and every new discovery of science whatsoever. Not until a newly-discovered method has passed the grand final examination of the general practitioner's average cases is it admitted to the rights and privileges of a recognised position in orthodox and accredited medicine.

The great and acknowledged leaders of medicine and surgery know all these things quite well, and it is because they are endowed with the real scientific competence which distinguishes between things that differ that they are leaders. It is not the real leaders, but the would-be great men who are constantly, like the advertising tradesman, puffing their own wares—that is themselves. It is a painfully sorry business, a business we are almost ashamed to write about. But the constant reiteration of such stale platitudes at hospitals, and especially at opening lectures, has raised them into articles of faith among the inexperienced; so much so, indeed, that at the present time the very foolishest Michaelmas goose of a first-year's man feels himself entitled to look down from the sublimest of elevations upon the mere general practitioner. Nay, even the nurse, who but yesterday was a milliner's apprentice, has learnt her lesson, and informs her superior with pert imbecility that the doctor at her last typhoid case was "only a general practitioner." Could stupidity further go?

The mature conviction of the present writer, who is not himself a general practitioner, is that there is no class in the medical profession which deserves better of the public than the faithful and conscientious class of general practitioners: and, what is more to our present purpose, there is no class more ardent and eagerin the pursuit of advancing medical science, or more competent to express a sound judgment upon the merits of the work of the laboratory and the hospital clinique. The general practitioner is in immediate contact with those sick persons who pay their own fees, and who are always resolved, as far as possible, to have "full value for their money" This cannot be said of either the laboratory scientist or the clinician in the hospital ward. The family doctor is compelled to be practical whether he will or no: to practically cure his patients, indeed, or to lose them to his more practical neighbour. For their own sakes, one would expect that the inexperienced junior members of the staff at our great hospitals would at least have worldly wisdom enough to keep silent, even though they might feel themselves to be so exceedingly superior to their more experienced brethren in general practice. But nothing can content them except holding themselves up at the expense of other practitioners to the admiration of raw students less experienced and more self-satisfied than even themselves. For shame, gentlemen! Prudence, if not courtesy, should restrain you!

